# Valorization of agro-industrial and forestry biomass ashes as soil amendments: integrating chemical screening, leaching dynamics, and *Beta vulgaris* L. response

**DOI:** 10.1038/s41598-026-58403-3

**Published:** 2026-06-18

**Authors:** Magdalena Oleksy-Sobczak, Andrzej Baryga, Łukasz Ściubak, Alina Kunicka-Styczyńska, Stanisław Brzeziński

**Affiliations:** Department of Sugar Industry and Food Safety Management, Faculty of Biotechnology and Food Science, Lodz University of Technology, Wólczańska 171/173, 90-530 Lodz, Poland

**Keywords:** Biomass ash valorization, Soil amendment, *Beta vulgaris* L., Trace elements, Nutrient recycling, Circular economy, Ecology, Ecology, Environmental sciences, Plant sciences

## Abstract

Sustainable management of biomass combustion ash is critical for circular economy transitions. This study provides a comprehensive evaluation of six biomass ashes (wood chips, forest residues, maize cobs, plum pits, sugar beet pulp, and distillery decoction) as potential soil amendments. Chemical characterization and leaching tests revealed high concentrations of Ca, K, Mg, and P, confirming significant agronomic potential. However, ashes from beet pulp and distillery decoction exceeded EU arsenic limits by up to 5.5-fold, with distillery decoction ash releasing 69% of its As during leaching. A pot experiment with *Beta vulgaris* L. demonstrated that ash application at 2–8 Mg ha⁻¹ consistently increased soil pH and enhanced fresh biomass by up to 59%, particularly when combining nutrient-rich and Ca-rich ashes. Nevertheless, high application rates of specific blended variants increased Pb and Cd uptake in roots, likely driven by dissolved organic carbon (DOC) mobilization. These findings indicate that while biomass ashes effectively correct soil acidity and improve crop yields, their valorization requires rigorous feedstock screening and dose control to ensure regulatory compliance and food safety.

## Introduction

Plant biomass is increasingly recognised as a key component of sustainable energy systems in the context of climate change mitigation, environmental degradation and the need to reduce greenhouse gas emissions. When produced sustainably, biomass combustion can effectively replace fossil fuels, as biogenic CO₂ emissions are largely offset by photosynthetic uptake. Its use is supported by European legislation promoting clean and renewable energy production^1,2^. At the same time, concerns related to indirect land‑use change (ILUC) have shifted attention toward second‑ and third‑generation feedstocks—agricultural and forestry residues, sewage sludge, organic waste fractions and algae—which do not compete with food production^3^.

The suitability of biomass as a fuel depends strongly on chemical composition, moisture content and combustion technology. High‑quality feedstocks such as wood chips offer stable, low‑emission combustion even in small‑scale systems^4^. Biomass currently accounts for a major share of renewable heat production, and global bioenergy generation is projected to exceed 3000 TWh yr⁻¹ by 2050^5^. However, increasing biomass use inevitably leads to substantial ash generation. In 2021, global biomass consumption of 3 Gt yr⁻¹ produced ~ 170 Mt of ash, and future consumption could generate close to 1000 Mt annually^6^. EU legislation^7^ therefore encourages valorisation of biomass ashes as secondary raw materials within circular‑economy frameworks.

Biomass ashes are typically alkaline and enriched in Ca, K, Mg and P, offering potential agronomic benefits including pH correction, improved soil structure and enhanced nutrient availability. Their incorporation has been shown to raise soil pH, increase micronutrient content and improve crop yields^8^. These benefits stem in part from mineral phase interactions, particularly the Ca–P system, which enhances buffering capacity and immobilizes trace elements^4,9^. Conversely, chloride and sulfate salts may increase ionic strength and promote metal mobilization^10,11^. However, the properties and environmental performance of biomass ashes are not uniform and depend strongly on the type of feedstock used for combustion. Woody biomass ashes, such as those derived from forest residues or wood chips, are typically characterised by high calcium content and strong liming potential, resulting in effective pH elevation and improved soil buffering capacity^8,12^. In contrast, ashes originating from agricultural residues and agro-industrial by-products, including straw, crop residues or food-processing wastes, often contain higher levels of potassium and phosphorus, which can enhance nutrient availability but may also increase solubility and leaching potential^4,6^. These compositional differences are further reflected in the environmental behaviour of biomass ashes in soil systems. The mobility and fate of nutrients and trace elements are controlled by ash mineralogy, pH, and the presence of soluble inorganic and organic fractions, which influence leaching processes and plant uptake. In particular, interactions between dissolved organic carbon and trace elements may enhance their transport and bioavailability under certain conditions, despite generally favourable pH-driven immobilisation mechanisms^10–13^. Nevertheless, despite the growing body of research, studies integrating physicochemical characterisation, leaching behaviour and plant uptake for diverse biomass ash types remain limited, particularly in the context of agro-industrial residues. Understanding these combined effects is therefore essential for assessing the safe and effective use of biomass ashes as soil amendments.

Despite their agronomic advantages, the large-scale agricultural use of biomass ash remains limited due to regulatory inconsistencies across the EU. While some Member States have established advanced recycling frameworks, others rely heavily on landfilling^10^. Environmental concerns include the potential leaching of Cd, Pb, Cr and Ni, which depends on total concentration, speciation, pH and particle size. Although many metals occur in residual, low‑mobility forms, exceptions such as As and Zn require careful assessment. Tools such as the Risk Assessment Code, Hakanson Index and sequential extraction methods support evaluation of metal mobility and environmental risk^11–13^.

Given the agronomic potential and the variability in composition and environmental behaviour among different biomass ashes, safe utilisation of biomass ash requires detailed characterisation, including pH, sorption properties and metal immobilisation capacity. Without appropriate management, uncontrolled application may threaten soil quality and human or animal health. The present study provides an integrated assessment of biomass ashes derived from selected agri‑food and forestry residues, combining chemical characterisation, environmental risk evaluation and a pot experiment with beetroot (*Beta vulgaris* L.). Unlike most previous studies focusing on individual aspects of biomass ash utilisation, this work integrates physicochemical characterisation, leaching behaviour, and plant response, providing a more comprehensive assessment of ash suitability for agricultural applications. The aim of this study was to determine the suitability of different ashes and their mixtures for agricultural use, with emphasis on soil pH, biomass production and nutrient or trace-element accumulation, thereby supporting evidence-based strategies for ash valorisation within circular-economy frameworks, particularly given the high variability observed among different ash types.

## Materials and methods

### Experimental design and potted plant experiment with *Beta vulgaris* L.

The study comprised three experimental stages: physicochemical characterization of biomass ashes, assessment of element mobility using leaching tests, and a pot experiment with *Beta vulgaris* L. All measurements were conducted in accordance with European and international standards (CEN/ISO) to ensure methodological consistency, reproducibility, and comparability.

The pot experiment was conducted in 2023 under outdoor conditions in a completely randomised design with three replicates per treatment. Soil from the 0–20 cm arable layer under winter wheat in eastern Wielkopolska (Poland) was air-dried, sieved (< 5 mm), homogenised and placed into pots (7 kg dry soil per pot). Three ash types were selected for the pot experiment based on the results of chemical characterisation and leaching tests: maize cob ash, wood-chip ash, and Ca-enriched ash produced by combusting biomass with 5% defecation lime (5% w/w). Two blended variants were also prepared: Mix (25% w/w maize ash + 75% w/w wood-chip ash) and Maize + Lime (50% w/w maize ash + 50% w/w Ca-enriched ash). The rationale for this selection is discussed in the section "Environmental implications of ash composition". Ash production and characterisation are described in the following subsection. Treatments included a control and five ash-based series, each applied at three doses (8, 16 and 32 g pot⁻¹), corresponding to 2, 4 and 8 Mg ha⁻¹. All pots received 1 g ammonium nitrate (34% N) and 1 g monoammonium phosphate (12% N, 52% P₂O₅). This corresponds to approximately 115 kg N ha⁻¹. Ashes and fertilisers were thoroughly mixed with soil before filling pots. Each pot was planted with five seedlings of *Beta vulgaris* L. subsp. *vulgaris*. Pots were placed outdoors, exposed to natural weather and irrigated manually to avoid water stress. Plants were harvested at physiological maturity (approximately 60–70 days after planting) on 9 October 2023. Roots were carefully removed from the soil and gently washed with deionised water to remove adhering particles. Fresh biomass of leaves, roots, and total plant material was measured. Plant tissues were then separated and either frozen at − 20 °C to preserve their chemical composition prior to analysis or dried at 60 °C to constant mass for determination of dry matter and subsequent grinding.

All measurements and chemical analyses of ashes, soils and plant tissues were performed in triplicate. Results are presented as mean values with standard deviation.

### Biomass materials and ash production

The study investigated six biomass types representing forestry residues (forest wood chips, sawmill wood chips), agricultural by‑products (maize cobs, plum pits) and agro‑industrial waste streams (sugar beet pulp, distillery decoction). All materials were sourced from local suppliers in Poland, mainly within the Łódź Voivodeship, ensuring short supply chains and consistent feedstock quality. Sampling took place in autumn 2023, directly after harvesting or processing, to reflect typical availability.

Forest wood chips were derived from sustainable thinning and maintenance operations in mixed hardwood–softwood stands (alder, birch, oak, pine, spruce), providing material of low mineral contamination and favourable combustion characteristics. Sawmill wood chips originated from wood-processing residues free of chemical additives and were highly homogeneous in terms of feedstock composition and particle size. Maize cobs were collected after maize (*Zea mays* L.) shelling and represent a commonly available post‑harvest by‑product. Plum pits (*Prunus domestica* L.) were obtained from fruit‑processing lines following mechanical pitting and constitute a dry, lignified, physically stable biomass fraction.

Among agro‑industrial residues, sugar beet pulp (from *Beta vulgaris* L. subsp. *vulgaris*) was sourced after sucrose extraction and mechanical pressing in a sugar factory, while distillery decoction originated from cereal‑based bioethanol production, primarily wheat (*Triticum aestivum* L.) and maize (*Zea mays* L.), and was collected after the distillation stage.

Prior to combustion and analysis, all biomass samples were air‑dried at 20–25 °C and 40–60% relative humidity to constant mass to minimise moisture variability and ensure comparability across feedstocks. The selected materials encompassed both lignin‑rich woody biomass and carbohydrate‑rich residues, allowing evaluation of how contrasting biochemical composition influences combustion properties and ash chemistry. Their selection reflects typical biomass resources available in Central Europe and their relevance for potential agricultural applications of the resulting ashes.

### Analytical methods

#### Biomass characterisation

The test for biomass was conducted for working state, which refers to fuel in its current form, delivered directly to the furnace. In this state, the material contains natural moisture and particle size characteristic of the process. Representative biomass samples were air‑dried under laboratory conditions (20–25 °C, 40–60% relative humidity) for several days until constant mass was reached, then milled to a particle size < 1.0 mm using a cutting mill SM 300 (Retsch GmbH, Haan, Germany), homogenised, and stored in airtight containers at room temperature prior to analysis.

##### Moisture content

Moisture content was determined according to PN‑EN ISO 18134‑1:2015‑11 and PN‑EN ISO 18134‑3:2015‑11. Approximately 1–2 g of sample was dried at 105 ± 2 °C in a forced‑air oven UF110 (Memmert GmbH, Schwabach, Germany) until constant mass (< 0.1% mass change). Moisture was expressed as the percentage mass loss.

##### Ash content and volatile matter

Ash content and volatile matter were determined using a high‑temperature muffle furnace L9/11/B410 (Nabertherm GmbH, Lilienthal, Germany). Ash content was analysed following PN‑EN ISO 18122:2016‑01 by combusting dried samples in Al₂O₃ crucibles at 550 ± 25 °C to constant mass. Volatile matter was assessed according to PN‑EN ISO 18123:2016‑01 by heating samples placed in covered crucibles at 900 ± 10 °C for 7 min. Results were expressed as % DM, with volatile matter corrected for moisture.

##### Calorific value

The gross calorific value (higher heating value, HHV) and net calorific value (lower heating value, LHV) were determined using an isoperibolic bomb calorimeter C 6000 (IKA‑Werke GmbH & Co. KG, Staufen, Germany) equipped with a C 6010 combustion vessel, in accordance with PN‑EN ISO 18125:2017‑07. Calibration was carried out using certified benzoic acid (NIST SRM 39j, LGC Standards, Germany). Results were reported in kJ kg⁻¹ and represent a direct measure of the energy potential of the biomass fuels.

##### Elemental composition (C, H, N, S, Cl)

Carbon (C), hydrogen (H) and nitrogen (N) were analysed by high‑temperature combustion using an elemental analyzer vario EL cube (Elementar Analysensysteme GmbH, Langenselbold, Germany) following PN‑EN ISO 16948:2015‑07. Samples were combusted in an oxygen-rich atmosphere at > 950 °C. Calibration was performed with certified reference materials (BBOT; LECO Corp., USA; Elemental Microanalysis, UK), and instrument performance was checked daily. Validated analytical ranges were C: 5.0–50.0%, H: 0.20–6.0%, N: 0.002–8.00%. Total sulfur (S) was determined according to PN‑EN ISO 16994:2016‑10 using high‑temperature combustion with infrared detection on a LECO SC832 analyzer (LECO Corporation, St. Joseph, MI, USA). The analytical range for S was 0.06–5.0%, with calibration linearity R² > 0.999 and recoveries 90–110%. For quality control, total nitrogen was additionally measured using the Kjeldahl method (PN‑EN 13342:2002). Samples were digested in H₂SO₄ Suprapur^®^ (Merck, Germany) with catalyst in a BÜCHI K‑439 unit and distilled/titrated on a BÜCHI K‑375. Chlorine (Cl) content was determined potentiometrically after nitric‑acid digestion according to internal procedure PB/FCH/89/A:2014 using a Metrohm 916 Ti‑Touch titrator with a 6.0502.150 chloride ISE. Calibration employed Certipur^®^ Cl standards (Merck, Germany). The analytical range was 0.02–1.0%.

#### Ash characterisation

Ashes were stored in airtight polyethylene containers at ambient conditions prior to analysis.

##### Dry matter and organic fractions

Dry matter (DM) was determined according to PN‑EN 15934:2013‑02 by drying at 105 ± 2 °C to constant mass in a forced‑air oven Memmert UF110 (Memmert GmbH, Schwabach, Germany). Total organic carbon (TOC; non-purgeable) was determined by high-temperature combustion with infrared detection using a Shimadzu TOC-L analyser equipped with IR detection (Shimadzu TOC‑L with solid‑sample module; Shimadzu Corp., Kyoto, Japan). Samples were combusted at high temperature in an oxygen-rich atmosphere, and the evolved CO₂ was quantified by infrared detection. Loss on ignition (LOI) and organic substances were measured as per PN‑EN 15935:2022‑01 at 550 ± 25 °C in a muffle furnace Nabertherm L9/11/B410 (Nabertherm GmbH, Lilienthal, Germany).

##### Major and trace elements in the solid phase

Major and trace elements were quantified after closed‑vessel aqua regia digestion (HCl: HNO₃ = 3:1, v/v) following PN‑EN 13657:2006 using a microwave digestion system Milestone ETHOS UP (Milestone Srl, Italy). Digests were diluted with ultrapure water (18.2 MΩ·cm) and filtered through 0.45 μm Sartorius membranes. Elemental analysis (P, Ca, Mg, Na, K, Cr, Cd, Cu, Ni, Pb, Zn, Hg, As) was performed by ICP‑OES according to PN‑EN ISO 11885:2009 using an Agilent 5110 VDV spectrometer. Calibration employed multi‑element Certipur^®^ standards (Merck, Germany) with Y/Sc as internal standards to correct for drift; linearity satisfied R² > 0.999. Mercury was additionally verified by CVAAS following PN‑EN ISO 12846:2012 + Ap1:2016‑07 using internal laboratory procedure PB/I/11/D:2020. Phosphorus and calcium were reported also as P₂O₅ (factor 2.29) and CaO, in accordance with PN‑EN 15961:2017‑02 and PN‑EN 15960:2011. Chloride (Cl⁻) was determined by potentiometric titration following PB/FCH/10/E:2017 using a Metrohm 916 Ti‑Touch titrator (Metrohm AG, Switzerland) with a combined Cl‑ISE. Water‑soluble sulfate (SO₄²⁻) was quantified by ion chromatography using Metrohm 940 Professional IC Vario according to PN‑ISO 11048:2002 and PN‑EN ISO 10304‑1:2009. Analyses were performed in triplicate, with procedural blanks and calibration‑verification standards run every ten samples. Matrix‑matched calibrations and certified reference materials (CRMs) for ash/fly ash were included for recovery control (acceptable recovery: 90–110%).

##### Particle size distribution

Particle size distribution, including the < 0.063 mm fraction, was determined according to PN‑R‑04032:1998 using a sieve shaker Retsch AS 200 (Retsch GmbH, Germany). Selected samples were additionally analysed by laser diffraction using a Malvern Mastersizer 3000 (Malvern Panalytical Ltd., UK).

#### Soil and plant analyses

Soil pH (1 M KCl), total nitrogen, and elemental composition of plant tissues were determined using the instruments, standards and QA/QC as described previously (biomass/ash characterisation and analytical protocols); the same laboratory equipment, calibration strategy (matrix-matched standards, R² > 0.999), replication and recovery criteria (typically 90–110%) were applied here.

#### Leaching tests

Element mobility from biomass ashes was evaluated using leaching tube tests conducted under controlled laboratory conditions with deionised water, following a standard leaching procedure commonly applied for the assessment of element mobility from solid inorganic materials. The applied procedure was intended for comparative assessment of element mobility rather than absolute environmental exposure modelling.

After extraction, leachates were filtered through 0.45 μm membrane filters and analysed for macroelements, anions and trace metals using validated analytical methods (ICP-OES and ion chromatography). Total element concentrations in the solid ashes were determined independently after aqua regia digestion. The degree of leaching presented in Table 3 was calculated for each element as the percentage of the total element content released into the aqueous phase according to the following relationship:

$$Leaching\left( \% \right) = \frac{{element~mass~in~leachate}}{{total~element~mass~in~ash}}x100$$

### Statistical analysis

Assumptions of normality and homogeneity of variance were verified prior to analysis using the Shapiro–Wilk test for normality and Levene’s test for homogeneity of variances. Statistical evaluation was performed using analysis of variance (ANOVA) at a significance level of *p* ≤ 0.05. A two-way ANOVA was applied to assess the effects of ash type, application rate and their interaction. When significant effects were detected, differences between treatments were identified using post-hoc comparisons. Different letters in tables and figures indicate statistically significant differences between means. Statistical analyses were carried out using Statistica v.13 (StatSoft, Poland).

## Results and discussion

### Physicochemical properties of biomass feedstocks and implications for combustion

The physicochemical and energy properties of biomass depend on numerous factors, including plant species, growth conditions, and supply chain practices^14^. Table 1 presents selected parameters affecting combustion efficiency and energy potential of the biomass, which are directly relevant for understanding the resulting ash composition. These parameters are presented to provide context for ash composition, which directly determines its behaviour as a soil amendment, including pH, nutrient content, and trace element mobility.

**Table 1:** General physicochemical characteristics of different types of biomass intended for combustion.

Tested parameters	Units per DM	Type of biomass					
		Wood chips	Forest wood chips	Distillery decoction	Plum pits	Beet pulp	Maize cob cores
Total moisture	%	35.0 ± 5.6^a^	20.1 ± 3.2^b^	72.9 ± 11.7^c^	24.1 ± 3.9^b^	84.0 ± 13.4^c^	18.5 ± 3.0^b^
Ash	%	< 0.50 ± 0.13^a^	0.64 ± 0.17^a^	4.94 ± 1.28^b^	7.86 ± 2.04^b^	< 0.50 ± 0.13^a^	0.72 ± 0.19^a^
Volatile matter	%	52.6 ± 7.4^a^	62.9 ± 8.8^a^	< 40.0 ± 5.6^b^	60.0 ± 8.4^a^	< 40.0 ± 5.6^b^	65.1 ± 9.1^a^
Carbon /C	%	48.7 ± 2.4^a^	40.3 ± 2.0^b^	50.7 ± 2.5^a^	38.0 ± 1.9^b^	7.2 ± 2.2^c^	36.2 ± 1.8^b^
Heat of combustion	kJ kg^− 1^	18 595 ± 558^a^	19 475 ± 584^a^	21 785 ± 654^b^	21 455 ± 644^b^	16 368 ± 491^c^	13 332 ± 400^d^
Calorific value	kJ kg^− 1^	10 770 ± 323^a^	14 520 ± 436^b^	18 420 ± 552^c^	15 170 ± 455^b^	< 7 000 ± 210^d^	9 737 ± 292^a^
Total sulfur	%	< 0.060 ± 0.012^a^	< 0.060 ± 0.012^a^	0.202 ± 0.042^b^	< 0.060 ± 0.012^a^	< 0.060 ± 0.012^a^	< 0.060 ± 0.012^a^
Hydrogen /H	%	4.09 ± 0.41^a^	4.96 ± 0.50^ac^	7.28 ± 0.60^b^	4.39 ± 0.44^a^	5.99 ± 0.60^c^	5.45 ± 0.54^c^
Chlorine	%	< 0.02 ± 0.09^a^	< 0.02 ± 0.09^a^	0.36 ± 0.12^b^	< 0.02 ± 0.01^a^	< 0.02 ± 0.01^a^	0.11 ± 0.04^c^
Total Kjeldahl nitrogen	%	0.208 ± 0.046^a^	0.418 ± 0.092^b^	1.450 ± 0.217^c^	0.261 ± 0.057^a^	1.520 ± 0.229^c^	0.411 ± 0.090^b^

value “<“ - result below the limit of quantification; DM – dry matter; a, b, c, d — statistically significant differences between biomass samples within the same tested parameter, *p* ≤ 0.05.

Total moisture content ranged from 18.5% (Maize cobs) to 84% (beet pulp). High moisture significantly reduces the heating value, as energy is consumed for water evaporation. This lowers combustion temperatures and increases emissions, corrosion risks, and transport costs. Biomass with 50–60% moisture typically yields a lower heating value of 5–8 MJ kg⁻¹ and requires specialised fluidised bed boilers. Conversely, partially dried biomass (15–20% moisture) can reach 15–17 MJ kg⁻¹^12^. Consequently, the most favourable calorific values were recorded for forest wood chips, plum pits, and maize cobs, which had moisture contents of approximately 20%.

Ash content varied from < 0.5% to 7.86% (Table 1). This is substantially lower than the 12–22% typical of hard coal, thereby reducing disposal requirements^15^. Although biomass ash melts at 700–900 °C—compared to > 1200 °C for coal—and potentially increases slagging and fouling risks, the minimal ash quantities produced make the studied feedstocks operationally attractive^16^. Furthermore, volatile matter in the samples ranged from 40% (distillery decoction, beet pulp) to 65.1% (maize cobs). While high volatile matter facilitates rapid ignition, it necessitates an extended gas combustion zone and strict temperature control to prevent the formation of tar and unburned compounds^17^.

The carbon content of the tested samples (7.24–50.72% DM) corresponded to calorific values between < 7.0 and 18.42 MJ kg⁻¹ DM (Table 1). Although these values are lower than coal, biomass combustion provides the advantage of biogenic carbon cycling^18^. Regarding undesirable elements, sulphur and chlorine were highly favourable in wood chips, forest residues, plum pits, and maize cobs (S < 0.06% DM, Cl < 0.02% DM). In contrast, elevated concentrations were observed in distillery decoction (S 0.202% DM, Cl 0.361% DM), beet pulp, and maize cobs. Because elevated sulphur and chlorine can accelerate high-temperature corrosion and increase acid-forming emissions, feedstocks with higher levels may necessitate blending strategies or dedicated boiler designs^12,19^.

Finally, nitrogen content critically influences combustion emissions due to the formation of nitrogen oxides (NOₓ), which are subject to strict environmental regulations, such as the Industrial Emissions Directive 2010/75/EU^1^. The analysed woody biomass and plum pits exhibited low nitrogen content (< 0.5%), indicating minimal NOₓ emission potential^20,21^. Although maize cobs presented a moderate risk (0.411% DM), significantly higher nitrogen levels were recorded in distillery decoction and beet pulp (> 1.4% DM). These elevated levels require advanced NOₓ reduction technologies, such as selective non-catalytic reduction (SNCR), alongside strict temperature control. Overall, the physicochemical profiles indicate that wood chips, forest residues, maize cobs, and plum pits are well-suited for standard boilers, whereas high-moisture and nitrogen-rich feedstocks necessitate pre-drying and specialised equipment.

### Characteristics of biomass ashes relevant to agricultural use

As biomass use in energy systems grows, ash generation is increasing accordingly, intensifying the need for sustainable management within circular-economy frameworks. Recent studies highlight that biomass-ash valorisation is essential for reducing environmental burdens and improving resource efficiency, with agricultural application as fertiliser or soil amendment identified as one of the most promising pathways^4,6^.

#### Chemical reaction, organic matter content and physical properties

Calcium amendments, including industrial by-products like sugar production defecation lime, are widely used to neutralise acidic soils and stabilise heavy metals. By elevating pH and enhancing sorption, calcium significantly reduces metal mobility and bioavailability^22^. Given these ameliorative properties, ash generated from the combustion of wood chips blended with 5% defecation lime was analysed to evaluate its potential as a soil amendment.

The ash quality assessment (Table 2) evaluated physicochemical properties critical for agricultural application, particularly organic matter content, which must be minimised to prevent odour and gaseous emissions during storage and field use. No significant odour-related issues are expected for the analysed ashes due to their low organic matter content. Both loss on ignition (LOI) and total organic carbon (TOC) were found to be low: LOI remained < 0.5% DM for most samples (reaching maximums of 2.9% DM for forest wood chips and 2.6% DM for maize cobs), while TOC ranged from < 0.5 to 3.4% DM. These values align with typical thresholds for agricultural biomass ashes (LOI < 5% DM), indicating negligible biodegradable matter and a low risk of putrefaction or emissions, such as NH₃ and H₂S^4,6^. Consistent with findings for industrial-scale ashes^23^, such low carbon contents confirm that the analysed ashes are sanitarily safe for handling and soil application.

**Table 2:** Selected quality parameters of the ashes originating from the combustion of various types of biomass.

Tested parameters	Units	Tested ashes						
		Wood chips	Forest wood chips	Distillery decoction	Plum pits	Beet pulp	Maize cob cores	Wood chips with lime
pH at 20 °C		3.8 ± 0.2^a^	12.4 ± 0.2^b^	7.9 ± 0.2^c^	8.3 ± 0.2^c^	12.9 ± 0.2^b^	11.1 ± 0.2^d^	6.9 ± 0.2^e^
Dry Matter Content	%	> 99.0 ± 9.9^a^	2.9 ± 0.3^b^	> 99.0 ± 9.9^a^	> 99.0 ± 9.9^a^	> 99.0 ± 9.9^a^	2.6 ± 0.3^b^	> 99.0 ± 9.9^a^
Grain fraction < 0.063 mm	%	1.50 ± 0.09^a^	9.95 ± 0.09^b^	3.60 ± 0.18^c^	0.50 ± 0.03^d^	12.30 ± 0.12^b^	3.80 ± 0.04^c^	3.30 ± 0.16^c^
Total Organic Carbon (C)	% DM	< 0.50 ± 0.10^a^	3.40 ± 0.70^b^	0.63 ± 0.13^a^	< 0.50 ± 0.10^a^	< 0.50 ± 0.10^a^	1.10 ± 0.20^c^	< 0.50 ± 0.10^a^
Loss on Ignition (LOI)	% DM	< 0.50 ± 0.05^a^	2.90 ± 0.30^b^	< 0.50 ± 0.05^a^	< 0.50 ± 0.05^a^	< 0.50 ± 0.05^a^	2.60 ± 0.30^b^	< 0.50 ± 0.05^a^
Total Kjeldahl Nitrogen	% DM	0.056 ± 0.012^a^	0.081 ± 0.018^a^	0.072 ± 0.016^a^	0.600 ± 0.013^b^	0.040 ± 0.088^a^	0.066 ± 0.015^a^	0.016 ± 0.004^c^
Ammonium Nitrogen/N-NH4	% DM	< 0.005 ± 0.002^a^	< 0.005 ± 0.002^a^	< 0.005 ± 0.002^a^	< 0.005 ± 0.002^a^	< 0.005 ± 0.002^a^	< 0.005 ± 0.002^a^	< 0.005 ± 0.002^a^
Total Phosphorus/P	mg·kg⁻¹ DM.	733 ± 73^a^	9 180 ± 918^b^	109 398 ± 10 000^c^	1 600 ± 160^d^	7 582 ± 758^b^	8 570 ± 857^b^	5 853 ± 585^d^
Phosphorus/P2O5	mg·kg⁻¹ DM	10 700 ± 642^a^	21 000 ± 1470	250 521 ± 7 515	5 540 ± 111	28 950 ± 579	19 600 ± 182	13 400 ± 204
Calcium/Ca	mg·kg⁻¹ DM	30 700 ± 3 068^a^	123 000 ± 12 339^b^	9 450 ± 945^c^	4 840 ± 484^d^	256 302 ± 4 571^e^	39 600 ± 3 960^a^	137 900 ± 13 792^b^
Calcium/CaO	% DM	4.29 ± 0.17^a^	17.30 ± 0.69^b^	19.50 ± 1.32^b^	0.68 ± 0,03^c^	> 20.00 ± 1.20^b^	5.54 ± 0.33^a^	2 710.00 ± 54.20^c^
Magnesium/Mg	mg·kg⁻¹ DM	1 810 ± 181^a^	11 500 ± 846^b^	47 750 ± 2 500^c^	801 ± 80^d^	40 873 ± 4 231^c^	7 480 ± 728^e^	6 237 ± 624^e^
Sodium/Na	mg·kg⁻¹ DM	< 100 ± 15^a^	451 ± 68^b^	63 000 ± 7 500^c^	< 100 ± 15^a^	11 600 ± 1 740^d^	635 ± 95^b^	1 139 ± 171^e^
Potassium/K	mg·kg⁻¹ DM	2 880 ± 240^a^	53 022 ± 1 000^b^	233 990 ± 1 000^c^	7 330 ± 633^d^	28 790 ± 1 000^e^	89 690 ± 1 012^f^	18 342 ± 1 086^g^
Chlorides/Cl	mg·kg⁻¹ DM	151 ± 30^a^	493 ± 74^b^	37 900 ± 1 200^c^	273 ± 41^a^	513 ± 76^b^	7 510 ± 711^d^	2 359 ± 136^e^
Water-Soluble Sulphates	mg·kg⁻¹ DM	13 200 ± 1 320^a^	3 110 ± 311^b^	23 100 ± 2 210^c^	222 ± 19^d^	24 590 ± 2 459^c^	2 820 ± 282^b^	1 221 ± 116^b^
Chromium/Cr	mg·kg⁻¹ DM	35.3 ± 5.3^a^	18.2 ± 2.7^b^	9.7 ± 1.4^c^	< 0.3 ± 0.1^d^	3.7 ± 0.5^e^	15.7 ± 2.4^b^	38.7 ± 5.8^a^
Cadmium/Cd	mg·kg⁻¹ DM	0.05 ± 0.01^a^	0.07 ± 0.01^a^	0.94 ± 0.09^b^	< 0.05 ± 0.01^a^	< 0.05 ± 0.01^a^	< 0.05 ± 0.01^a^	4.66 ± 0.47^c^
Copper/Cu	mg·kg⁻¹ DM	< 0.40 ± 0.04^a^	68.80 ± 6.90^b^	63.90 ± 6.40^bd^	< 0.40 ± 0.04^a^	6.67 ± 0.67^c^	46.20 ± 4.60^d^	37.09 ± 3.71^d^
Nickel/Ni	mg·kg⁻¹ DM	< 1.00 ± 0.10^a^	13.80 ± 1.40^b^	36.40 ± 3.60^c^	< 1.00 ± 0.10^a^	1.25 ± 0.12^a^	9.58 ± 0.96^d^	7.41 ± 0.74^d^
Lead/Pb	mg·kg⁻¹ DM	< 1.00 ± 0.10^a^	5.61 ± 0.56^b^	26.50 ± 2.60^c^	< 1.00 ± 0.10^a^	< 1.00 ± 0.10^a^	2.73 ± 0.27^d^	16.58 ± 1.66^e^
Zinc/Zn	mg·kg⁻¹ DM	37.6 ± 5.6^a^	807.0 ± 121.2^b^	4 440.1 ± 666.2^c^	2.5 ± 0.4^d^	80.7 ± 12.1^e^	535.0 ± 80.0^f^	566.0 ± 84.9^f^
Mercury/Hg	mg·kg⁻¹ DM	< 0.05 ± 0.01^a^	< 0.05 ± 0.01^a^	0.10 ± 0.02^b^	< 0.05 ± 0.01^a^	0.06 ± 0.01^a^	< 0.05 ± 0.01^a^	< 0.05 ± 0.01^a^
Arsenic/As	mg·kg⁻¹ DM	1 823.0 ± 151.0^a^	4.8 ± 0.7^a^	274.0 ± 41.0^c^	< 5.0 ± 0.7^d^	84.8 ± 127.2^e^	< 5.0 ± 0.7^d^	< 5.0 ± 0.7^d^

value “<“ - result below the limit of quantification; value “>” - result above the limit of quantification, DM – dry matter; a, b, c, d, e, f — statistically significant differences between biomass samples within the same tested parameter, *p* ≤ 0.05.

The analysed ashes exhibited a broad pH range (Table 2), reflecting variations in feedstock mineral composition. Strongly alkaline values (pH 11.1–12.9) were observed for ashes from forest wood chips, beet pulp, and maize cob cores. Such alkalinity is consistent with Ca- and K-rich biomass, indicating a high neutralisation potential for acidic soils. Many scientific publications indicate that pH is a key controlling factor in metal mobility, with acidic conditions enhancing the solubility of metals such as Cu, Cd and Ni, whereas higher pH generally limits their mobility^13,24^. In contrast, wood chip ash displayed an acidic pH (3.8), which characterises materials poor in alkaline earth metals and limits their neutralising capacity^6^. Although biomass ashes are typically alkaline, such deviations toward acidic pH values may occur due to specific feedstock composition, incomplete combustion, or the presence of acidic mineral phases. This variability has been reported in previous studies on low-alkali or contaminated biomass fractions, particularly where mineral composition limits the formation of CaO-rich ash^6,14^. However, the addition of 5% defecation lime during wood chip combustion neutralised the resulting ash (pH 6.9). Antonkiewicz et al.^22^ demonstrated that calcium-rich biomass ashes increase pH to alkaline levels, simultaneously enhancing their liming value and buffering capacity. Likewise, Thek and Obernberger^12^ reported that ashes with elevated CaO contents (> 10–20% DM) exhibit significantly higher neutralisation potential compared to untreated wood ashes.

#### Macronutrient composition of biomass ashes

Furthermore, the analysed ashes were identified as a valuable source of macronutrients essential for plant growth and soil amelioration (Table 2). Calcium (Ca), vital for soil structure improvement and root system development, reached the highest levels in beet pulp ash (256 302 mg kg⁻¹) and in wood ash enriched with defecation lime (137 900 mg kg⁻¹). Potassium (K), crucial for plant water balance and stress resistance, was most abundant in distillery decoction ash (233 990 mg kg⁻¹) and maize cob ash (89 690 mg kg⁻¹). Phosphorus (P), essential for root and flower development, and magnesium (Mg), a key element in photosynthesis, exhibited their highest concentrations in distillery decoction ash (109 398 mg kg⁻¹ and 47 750 mg kg⁻¹, respectively), with Mg also prominent in beet pulp ash (40 873 mg kg⁻¹). Converted to mass percentages, these contents correspond to 0.01–10.9% P, 0.29–23.4% K, 0.48–25.6% Ca, and up to 4.8% Mg. These ranges align with findings by Stankowski et al.^25^, who reported average contents of 1.3% P, 3.4% K, 12.1% Ca, and 1.6% Mg (DM) for industrial biomass ashes. Such high nutrient concentrations highlight the potential of the investigated ashes as soil amendments, provided that potentially toxic elements are carefully controlled.

#### Heavy metal content of biomass ashes in relation to regulatory thresholds

Biomass combustion residues (European Waste Catalogue code 10 01 99) may be applied agriculturally, provided they meet the Commission Regulation (EU) 2019/1009^26^ contaminant limits for mineral fertilisers: 50 mg kg⁻¹ for arsenic (As) and cadmium (Cd), 140 mg kg⁻¹ for lead (Pb), and 2 mg kg⁻¹ for mercury (Hg). Accordingly, the analysed ashes were evaluated for heavy metal compliance (Table 2).

The analysis revealed considerable variability, particularly for As, which ranged from < 5.00 mg kg⁻¹ DM to levels significantly exceeding the 50 mg kg⁻¹ threshold. Specifically, ashes derived from distillery decoction and beet pulp (84.8 mg kg⁻¹ DM) exceeded the limit by approximately 5.5-fold and 1.7-fold, respectively. These results are consistent with those obtained by Adamczyk et al.^27^, who reported As levels in sunflower husk ash up to ten times the regulatory limit. The observed arsenic enrichment points to specific contamination pathways inherent to agro-industrial processing, particularly during drying stages. As demonstrated by Wu et al.^28^, As can be introduced into beet pulp during direct drum drying using combustion gases from coal or heavy oil. Under these conditions, volatile arsenic species (primarily As_2_O_3_) present in fossil fuel emissions can undergo surface deposition and chemisorption onto the organic matrix of the pulp. Similarly, the elevated Zn levels in distillery decoction may stem from the concentration effect during the evaporation or from the corrosion of galvanized processing equipment. To mitigate these risks and ensure compliance with EU 2019/1009^26^ standards, industrial transitions toward indirect steam-drying systems or the use of cleaner auxiliary fuels are recommended. Such technological adjustments would prevent the partitioning of flue-gas contaminants into the biomass, thereby preserving the quality of resulting ashes for safe agricultural valorization within circular resource systems.

Conversely, all examined ashes complied fully with Cd, Pb, and Hg regulations. Cadmium (< 0.050–4.658 mg kg⁻¹ DM) and lead (< 1.00–16.58 mg kg⁻¹ DM) concentrations reached their maximums in wood chips blended with defecation lime, yet remained well below permissible limits (Table 2). These low Cd values align with findings by Pazalja et al.^29^, who reported an average cadmium concentration of 0.56 mg kg⁻¹ (d.w.) in wood pellet ashes. The low Pb concentrations contrast with the elevated levels (up to 1,640 mg kg⁻¹) reported by Smołka-Danielowska and Jabłońska^30^ in fine ash fractions, which they linked to urban atmospheric deposition or secondary contamination during transport. Mercury concentrations were also negligible (< 0.005–0.10 mg kg⁻¹ DM), peaking in distillery decoction ash but remaining roughly 20 times below the threshold. This supports Vassilev et al.^31^, who noted that Hg predominantly volatilises during combustion, leaving only trace amounts in the ash. Ultimately, while the investigated ashes are safe regarding Cd, Pb, and Hg, the elevated As in specific feedstocks highlights the necessity of rigorous contaminant monitoring prior to agricultural application.

#### Effect of defecation lime addition on ash chemical composition

The addition of 5% (w/w) defecation lime to wood chips modified specific ash chemical properties, whereas core indicators (dry matter, total organic carbon, loss on ignition, and ammonium nitrogen) remained unchanged compared to untreated wood chip ash (Table 2). Total Kjeldahl Nitrogen decreased by approximately 70%, suggesting enhanced thermal decomposition and dilution by the calcium-rich additive. Conversely, total calcium content increased over four-fold, despite a reduction in CaO expressed as oxide, indicating that calcium was redistributed among different mineral phases (e.g. carbonates or sulfates) rather than occurring predominantly as CaO.

Water-soluble sulphates decreased by nearly an order of magnitude, and arsenic fell below the detection limit, indicating a strong mitigation of mobile oxyanion-forming species. In contrast, total concentrations of macronutrients such as phosphorus, potassium, magnesium, and sodium, as well as chlorides, increased several-fold, reflecting substantial enrichment by the lime additive. The fine particle fraction (< 0.063 mm) approximately doubled due to increased mineral fragmentation. Furthermore, trace elements (Cr, Cd, Cu, Ni, Pb, Zn) exhibited moderate increases relative to pure wood chip ash. These findings are consistent with the mechanisms described by Chaloupková et al.^32^, who demonstrated that calcium-based additives modify ash mineralogy and promote trace-element retention during co-combustion. Additionally, Kalembkiewicz et al.^14^ noted that industrial lime may contain minor metal impurities, underscoring the necessity for strict feedstock control. Ultimately, these results suggest that post-combustion blending is preferable to in-furnace addition for agricultural applications, as it allows for compositional tuning while effectively managing trace-metal risks.

#### Leaching behaviour of biomass ashes

Leaching of heavy metals remains a primary environmental concern for biomass ash application, as mobility is governed by speciation, pH, and particle size^13,24^. While acidic conditions typically enhance metal migration, natural processes like hydration and carbonation promote the immobilization of elements such as Cd, Cu, Ni, and Zn^33^. In this study, most ashes exhibited negligible leaching, complying with the Polish Regulation^34^, and thus indicating low environmental risk in terms of potential contamination of soil and groundwater under typical agricultural conditions (Table 3). However, selected agro-industrial residues, including distillery decoction ash and lime-modified variants, showed elevated leaching of specific elements such as As and Zn. Importantly, for the ash variants included in the pot experiment, higher mobility of soluble fractions in the leaching tests is consistent with the changes observed in plant uptake and post-harvest soil chemistry (see Sect. "Environmental implications of ash composition").

**Table 3:**
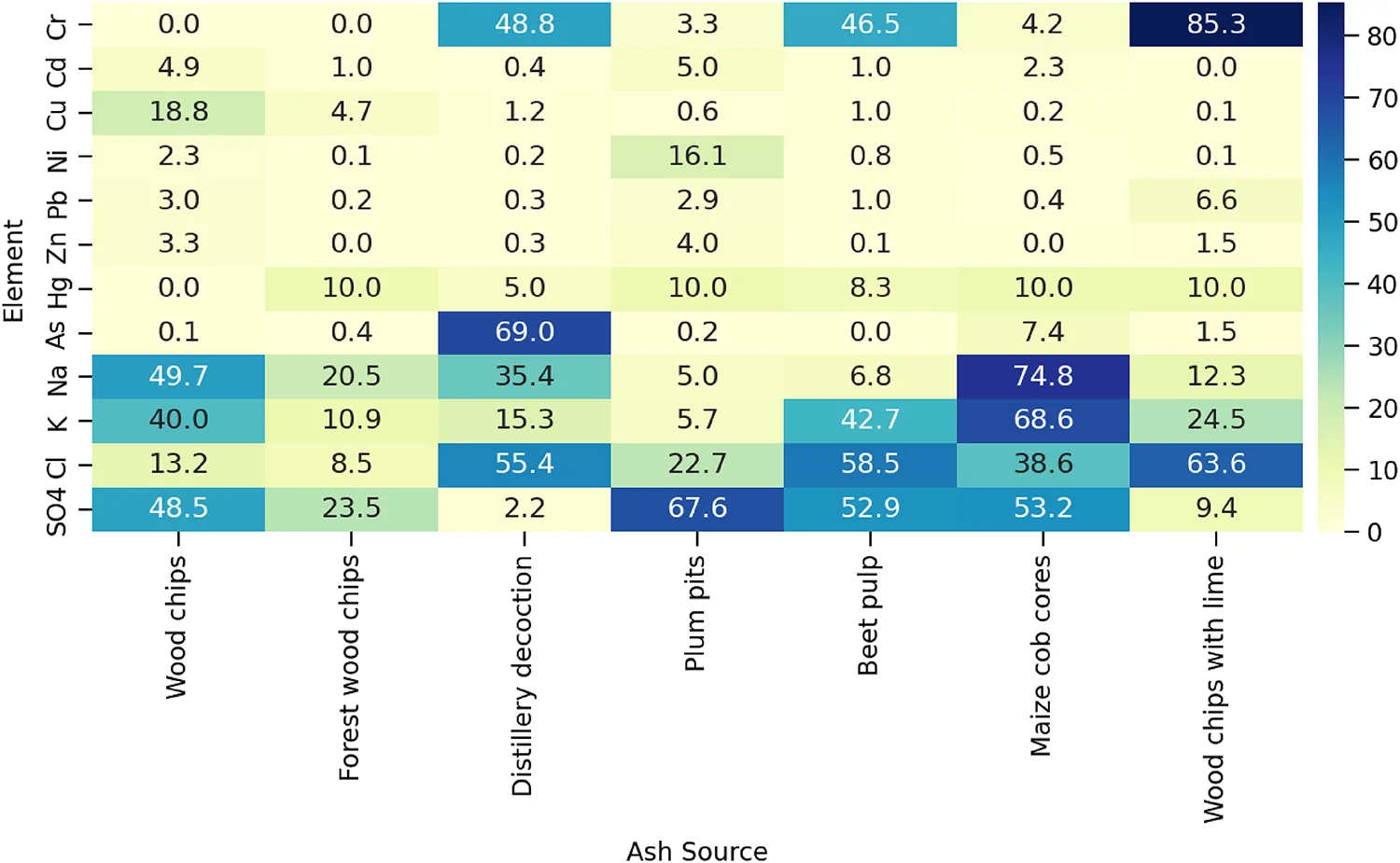
Degree of leaching elements from the analyzed ashes.

Leaching degrees varied significantly depending on the feedstock. The degree of leaching presented in Table 3 was calculated as the percentage of total element content released into the aqueous phase, as described in the "Materials and methods" section. For instance, Ni leaching was nearly zero in forest chips (0.07%) but reached 16.1% in plum pits (Table 3). Similar variability in leaching behaviour was reported by Drljača et al.^33^ who demonstrated that although total metal concentrations in biomass ashes may remain within regulatory limits, their mobility varies considerably depending on ash composition, pH and environmental conditions. In particular, non-woody residues tend to exhibit higher proportions of water-soluble or weakly bound fractions, leading to increased leaching potential.

Notably, the highest Cr mobility was recorded in wood-chip ash with lime (85.3%), contrasting sharply with pure wood-chip ash (0.03%), which suggests that lime addition may alter the mineral matrix. This observation is consistent with Ottosen and Sigvardsen^24^, who demonstrated that alkaline conditions and mineral transformations during ash ageing influence chromium speciation, promoting the oxidation of less mobile Cr(III) into highly mobile and toxic Cr(VI) forms. This process increases chromium solubility and its release into the aqueous phase, significantly enhancing its environmental mobility and potential risk for soil and groundwater systems.

Furthermore, Cu showed elevated mobility in untreated wood ash (18.75% vs. <5% in others), likely due to its low pH (3.8). These results are consistent with previous studies indicating that pH is a key controlling factor in metal mobility, with acidic conditions enhancing the solubility of elements such as Cu, Cd and Ni, whereas higher pH generally limits their mobility^13,24^. This mechanism is consistent with the elevated Cu leaching observed in the low-pH wood ash in the present study.

High arsenic release during leaching was observed for distillery decoction ash (69.0% of total As released), and elevated Zn leaching was noted for lime-blended variants (Table 3). Furthermore, You et al.^13^ demonstrated that most trace elements in biomass ashes from power plants occur predominantly in stable residual fractions, resulting in low mobility and limited bioavailability. In contrast to these generally stable matrices, the high leaching degree observed in this study for As indicates the presence of more labile and soluble forms, particularly in agro‑industrial residues such as distillery decoction and beet pulp, which exhibited the highest leaching values. This behaviour may be associated with a higher proportion of water-soluble components or weaker binding to mineral phases, although further structural analysis would be required to confirm the exact mechanisms. Consistent with these findings, distillery decoction ash exceeded the strict 50 mg kg⁻¹ threshold for arsenic established by Commission Regulation (EU) 2019/1009, surpassing the limit by approximately 5.5-fold, while sugar beet pulp ash exceeded it by 1.7-fold^26^. The elevated arsenic levels may be linked to upstream industrial processing. Arsenic enrichment in beet pulp can occur during direct drum drying via the deposition of volatile arsenic species (primarily As₂O₃) from combustion gases, while increased Zn levels in distillery decoction may result from concentration effects during evaporation or corrosion of processing equipment^28^. Importantly, these results indicate that agro-industrial residues with elevated arsenic levels require careful selection and management, including potential stabilisation or blending with low-contamination materials, to reduce leaching and ensure safe agricultural application.

Beyond metals, high leaching of salt fractions (Cl⁻ and SO₄²⁻ up to 63.6% and 67.6%) and alkali metals (K up to 68.6%) was observed. This indicates that the primary environmental impact of large-scale application may be driven by soil solution ionic strength rather than trace element toxicity.

Crucially, this pattern is consistent with the potential role of dissolved organic carbon (DOC) in trace element transport, particularly in organic-rich amendments. Previous studies indicate that DOC can form soluble metal–organic complexes, enhancing the mobility and bioavailability of metals such as Pb and Cd^13,32,35^. In our study, the increased root uptake of Pb and Cd at higher application rates of specific blends may be linked to DOC-mediated complexation, although DOC was not directly measured and this mechanism should therefore be interpreted as a plausible explanation rather than a confirmed cause.

By bridging these dimensions, this study provides an integrated perspective linking leaching dynamics, regulatory compliance, and plant uptake. The results indicate that evaluating biomass ash suitability should not rely solely on total metal concentrations, as environmental risk is influenced by element mobility, speciation, and interactions with organic fractions. From a regulatory perspective, this supports complementing total-content compliance with mobility-based screening and, where relevant, bioassays, particularly for materials containing higher organic fractions^13,32^.

### Environmental implications of ash composition

A major challenge in valorising biomass combustion residues is their chemical variability and the necessity to verify agronomic safety. To minimise trace-metal risks while maintaining functional contrasts, the experimental set was narrowed to three ashes: maize cob, wood chip, and wood chip co-combusted with defecation lime. Despite attractive macronutrient profiles, alternatives like distillery decoction and beet pulp were excluded due to elevated arsenic or zinc levels and higher leachability, which compromised regulatory compliance and environmental safety. The selected trio reflects realistic feedstock availability and provides a practical gradient of nutrients and alkalinity, ranging from an agro-residue rich in potassium, phosphorus, and magnesium, to a calcium-rich woody ash and a lime-enhanced variant with high neutralising capacity.

Beetroot (*Beta vulgaris* L.) was selected as the model crop due to its sensitivity to soil acidity and well-documented responses to pH correction and base-cation nutrition. Its taproot rapidly reflects changes in the availability of calcium, magnesium, and potassium, which are strongly influenced by ash additions and known to alter beetroot mineral profiles^34^. Furthermore, crops grown on acidic soils generally respond positively to liming and ash amendments through improved nutrient availability. Because *B. vulgaris* also exhibits distinct changes in micronutrient and potential trace-element uptake, it serves as a robust bioindicator for the joint assessment of both the agronomic benefits and environmental safety of ash-based conditioners^36^.

#### Impact of biomass ashes on soil chemical properties

It should be noted that the present study evaluated total element concentrations rather than extractable (plant-available) fractions. Therefore, interpretations of nutrient availability are based on indirect evidence, including plant response and soil chemical changes.

**Fig. 1:**
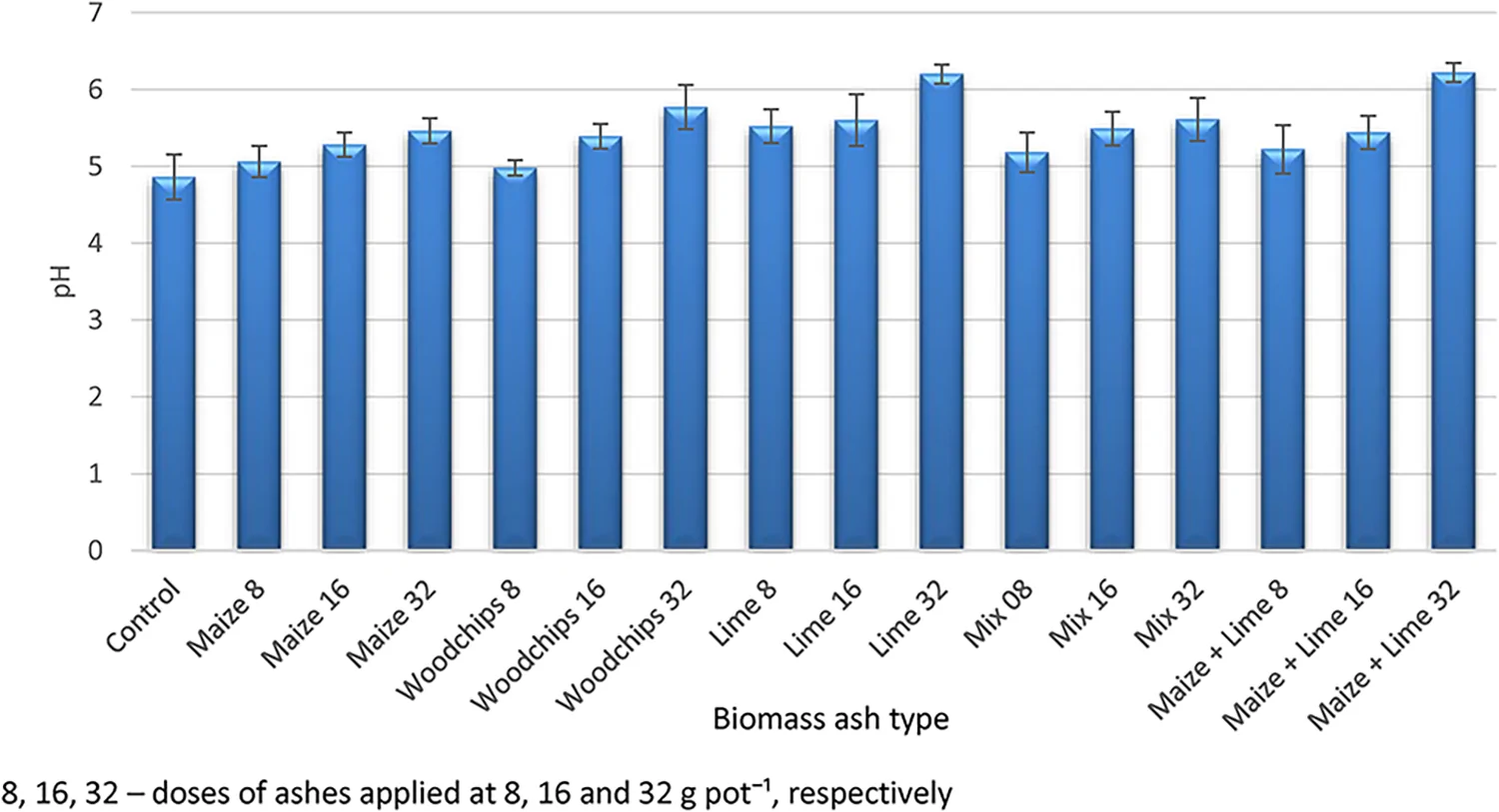
The effect of biomass ash type application on soil pH. Error bars represent standard deviation (*n* = 3).

Biomass ash significantly increased soil pH compared to the acidic control (pH 4.86) in a dose-dependent manner (*p* < 0.05; Fig. 1). The Ca-enriched ash produced the strongest liming response (pH 5.52–6.20), followed by wood-chip ash, mixed ashes, and maize cob ash. This sequence confirms that short-term soil neutralization depends on the application dose and the neutralizing capacity of reactive phases (such as CaO, MgO, and CaCO₃), rather than the initial pH of the ash slurry^36^. These outcomes align with Szatyłowicz and Hawrylik^8^, who demonstrated that biomass wood ash effectively performs as an agricultural liming agent, improving cation-exchange capacity and soil buffering. Agronomically, the maximum dose of Ca-enriched ash (8 Mg ha⁻¹) elevated soil pH to approximately 6.2. This threshold approaches the physiological optimum for *Beta vulgaris* L. (pH 6.0–6.8), which is essential to enhance phosphorus levels and reduce aluminum (Al) and manganese (Mn) toxicity.

**Fig. 2:**
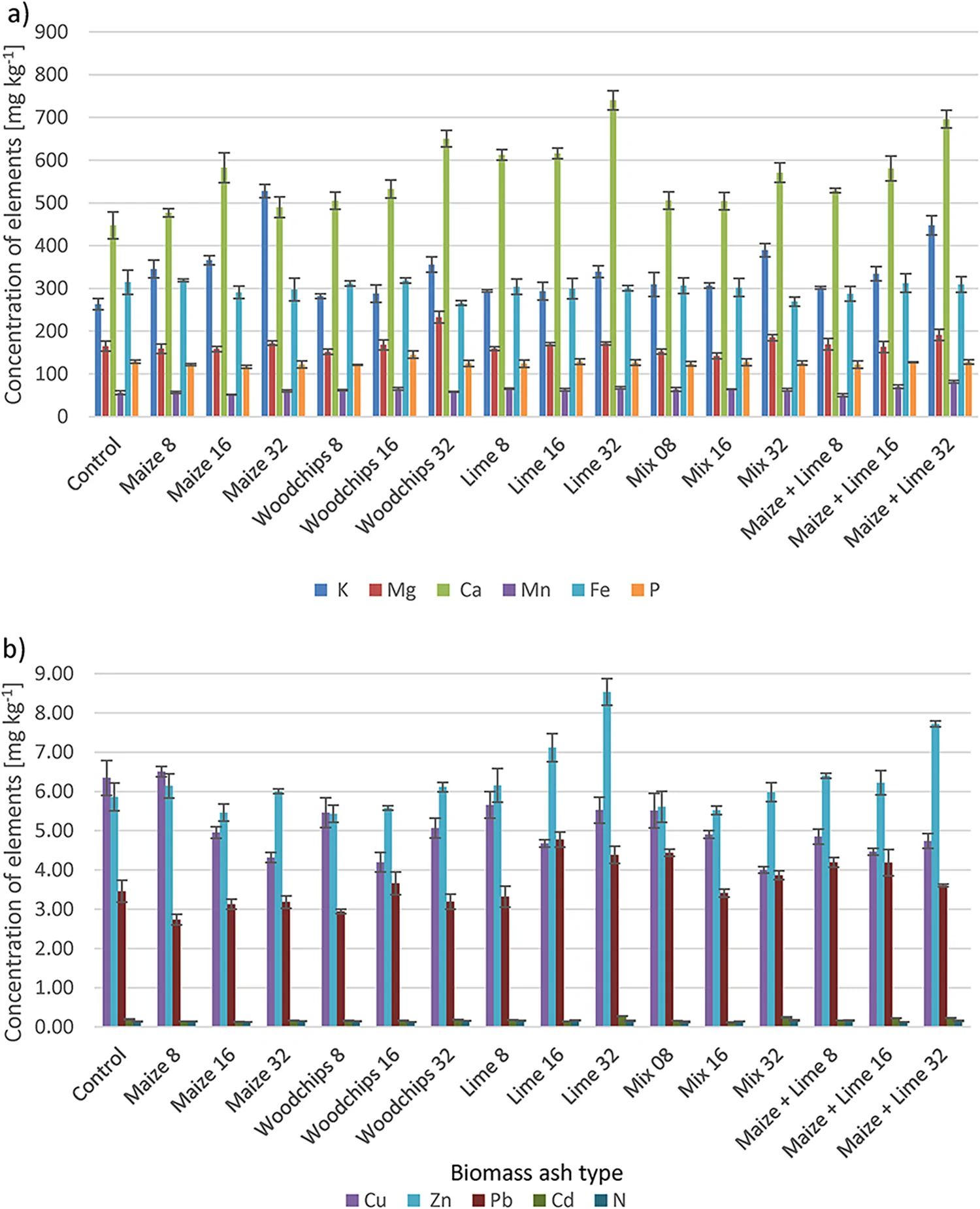
Effect of ash application on the accumulation of selected elements in soil, (**a**) macronutrients, (**b**) trace elements. Error bars represent standard deviation (*n* = 3).

Regarding soil microelement concentrations, lime-based treatments caused the most pronounced shifts in soil, yielding 8.9 mg kg⁻¹ Cu and 7.5 mg kg⁻¹ Zn at 32 t ha⁻¹ (Fig. 2). Such temporal variability in Cu and Zn concentrations does not necessarily indicate long-term accumulation, as element behaviour in soil is governed by mineral phases and pH-dependent sorption processes^4^. Conversely, potentially toxic trace metals (Pb and Cd) remained consistently low across all treatments. The stable soil Cd levels (0.05–0.1 mg kg⁻¹) were comparable to the control and to soils amended with uncontaminated forestry ashes, confirming that these clean-sourced biomass materials meet agricultural safety standards^31^. However, while total soil accumulation remained safe, the risk of localized metal mobilization at maximum doses must be considered in relation to dissolved organic carbon (DOC) dynamics, which can form soluble organo-metal complexes and alter root uptake.

Macroelements exhibited even stronger responses to ash application. Specifically, co-applying maize cob ash and defecation lime at 8 Mg ha⁻¹ elevated soil Ca levels to ~ 700 mg kg⁻¹ and K to ~ 300 mg kg⁻¹—the highest recorded values compared to the control baselines of ~ 450 and ~ 260 mg kg⁻¹, respectively (Fig. 2). This enrichment mirrors the typical elemental composition of biomass ash, which is dominated by calcium and alkali/alkaline earth metals^25^. Concurrently, pure wood-chip ash at 4 and 8 Mg ha⁻¹ produced the strongest increase in soil magnesium (~ 180 mg kg⁻¹ vs. ~120 mg kg⁻¹ in the control), due to the Mg-rich mineral nature of woody feedstocks^31^. Total soil nitrogen remained unchanged (Fig. 2) due to complete thermal volatilization during combustion, confirming that these ashes function as sources of base cations rather than nitrogen fertilizers^20,21^. Overall, the 8 Mg ha⁻¹ blends of maize ash with either defecation lime or wood-chip ash yielded the most balanced nutrient profiles, characterised by increased levels of macronutrients (Ca, K, Mg) while maintaining low concentrations of potentially toxic trace elements (Pb and Cd). These results highlight the agronomic benefit of blending contrasting ash feedstocks to optimize nutrient supply, prevent ion antagonism, and minimize environmental risks.

#### Beetroots response to ash application

Consistent with the factorial design, plant responses reflected both the independent and combined effects of ash type and application rate. Biomass ash application significantly increased dry matter of beetroot compared to the control (Table 4), confirming a true growth response rather than changes in tissue water content. The magnitude of this effect depended on the amendment type and dose. The strongest response was observed for the mixture of maize cob ash and wood-chip ash at 8 Mg ha⁻¹ (32 g pot⁻¹). Fresh biomass showed a similar trend, with total fresh yield increasing by ~ 59%, reflecting concurrent increases in both leaf and root mass.

**Table 4:** The effect of ash fertilizer type and dose on beetroot productivity in the pot experiment.

Ash Fertilizer	Dose (g pot⁻¹)	Fresh weight (g pot⁻¹) ± SD			Dry weight (g pot⁻¹) ± SD		
		Leaves	Roots	Total	Leaves	Roots	Total
Control	-	137.10 ± 2.74^a^	65.40 ± 2.61^a^	202.51 ± 2.02^a^	24.62 ± 0.98^a^	10.24 ± 0.60^a^	34.64 ± 2.42^a^
Maize	8	154.53 ± 7.72^ab^	74.47 ± 5.95^a^	228.91 ± 11.44^ab^	24.78 ± 0.49^a^	10.36 ± 0.30^a^	35.06 ± 2.10^a^
	16	139.90 ± 12.59^a^	68.12 ± 4.08^a^	207.95 ± 18.71^a^	25.70 ± 0.25^ab^	9.32 ± 0.18^b^	35.43 ± 2.80^a^
	32	169.30 ± 5.08^b^	84.52 ± 3.38^b^	253.81 ± 15.22^b^	24.72 ± 0.24^a^	11.58 ± 0.23^c^	36.21 ± 1.08^a^
Woodchips	8	141.11 ± 2.82^a^	87.62 ± 1.75^b^	228.78 ± 6.86^ab^	23.86 ± 1.42^a^	11.42 ± 0.34^c^	35.20 ± 1.76^a^
	16	136.44 ± 10.91^a^	80.17 ± 5.60^ab^	216.46 ± 12.98^a^	20.34 ± 1.82^a^	9.48 ± 0.18^b^	29.77 ± 1.18^b^
	32	167.80 ± 10.06^b^	93.92 ± 7.51^bc^	261.75 ± 5.23^bc^	27.11 ± 2.16^b^	12.08 ± 1.08^cd^	39.10 ± 3.12^a^
Lime	8	157.86 ± 12.62^ab^	89.83 ± 2.69^b^	247.63 ± 17.33^ab^	26.40 ± 1.05^ab^	11.21 ± 0.33^c^	37.66 ± 1.12^a^
	16	154.76 ± 4.64^ab^	89.72 ± 1.79^b^	244.42 ± 14.66^ab^	25.63 ± 2.30^ab^	11.11 ± 0.33^c^	36.76 ± 2.20^a^
	32	163.70 ± 4.91^ab^	93.83 ± 5.62^bc^	257.51 ± 2.57^b^	25.65 ± 0.51^ab^	11.47 ± 0.79^c^	37.00 ± 0.37^a^
Mix	8	134.61 ± 4.03^a^	75.26 ± 3.00^a^	209.89 ± 2.09^a^	22.83 ± 0.68^a^	11.66 ± 0.92^c^	34.40 ± 0.68^a^
	16	177.36 ± 15.95^bc^	107.84 ± 4.31^c^	285.16 ± 8.55^d^	22.90 ± 2.06^a^	13.50 ± 0.13^d^	36.43 ± 2.91^a^
	32	188.90 ± 13.22^c^	132.80 ± 6.64^d^	321.77 ± 22.51^d^	29.52 ± 0.59^b^	17.41 ± 1.56^e^	46.99 ± 0.93^c^
Maize + Lime	8	156.27 ± 9.37^ab^	93.51 ± 5.61^bc^	249.79 ± 22.47^abc^	24.21 ± 0.48^a^	12.36 ± 1.10^cd^	36.50 ± 2.55^a^
	16	169.43 ± 3.38^b^	111.32 ± 7.79^c^	280.70 ± 16.84^d^	24.97 ± 1.74^a^	14.62 ± 0.29^d^	39.52 ± 1.18^a^
	32	151.12 ± 10.57^ab^	113.27 ± 6.79^c^	264.32 ± 23.78^bcd^	23.65 ± 2.12^a^	13.57 ± 0.13^d^	37.10 ± 1.11^a^

a, b, c, d — statistically significant differences between doses within the same ash fertilizer and tested parameter, *p* ≤ 0.05.

Dry matter production followed a similar pattern, although the increase was slightly lower (~ 36%), indicating that the observed biomass accumulations were associated with actual plant growth rather than mere tissue water retention. These results are consistent with those obtained by Enesi et al.^37^, who demonstrated that biomass ash application enhances crop yield primarily through soil pH correction and the increased availability of base cations.

Conversely, wood-chip ash applied at 4 Mg ha⁻¹ (16 g pot⁻¹) reduced total dry matter despite a slight increase in fresh biomass. This behaviour may be associated with limited availability of nutrients released from woody biomass ashes at low doses, as well as local changes in soil pH. Compared to agro-industrial residues, woody ashes generally release nutrients more slowly, which may result in less uniform distribution in the soil solution and temporary imbalances in plant nutrition. Under such conditions, nutrient uptake efficiency may be reduced, particularly in systems with limited buffering capacity.

Liming alone resulted in a moderate but consistent increase in both fresh and dry biomass (~ 6–9%), primarily through soil acidity correction and improved Ca availability. Notably, the combined application of maize ash and defecation lime produced a stronger effect than either amendment applied separately. This distinct agronomic benefit confirms a synergistic interaction between nutrient supply and liming. A similar strategic enhancement of fertilization potential through combined matrices was recently highlighted by Rolka et al.^38^, who demonstrated substantial biomass and yield improvements in maize cultivation by co-applying woody biomass ash with organic digestate. While their approach optimized nitrogen mineralization and phosphorus availability through organic-mineral synergy, our study achieves a comparable agronomic boost in *Beta vulgaris* L. via an ash–lime matrix. This indicates that tailoring biomass ash with structural alkaline or organic components may represent a promising strategy to promote plant growth and neutralize soil acidity, provided that application rates are controlled to minimize risks of increased ionic strength^6,39^.

**Fig. 3:**
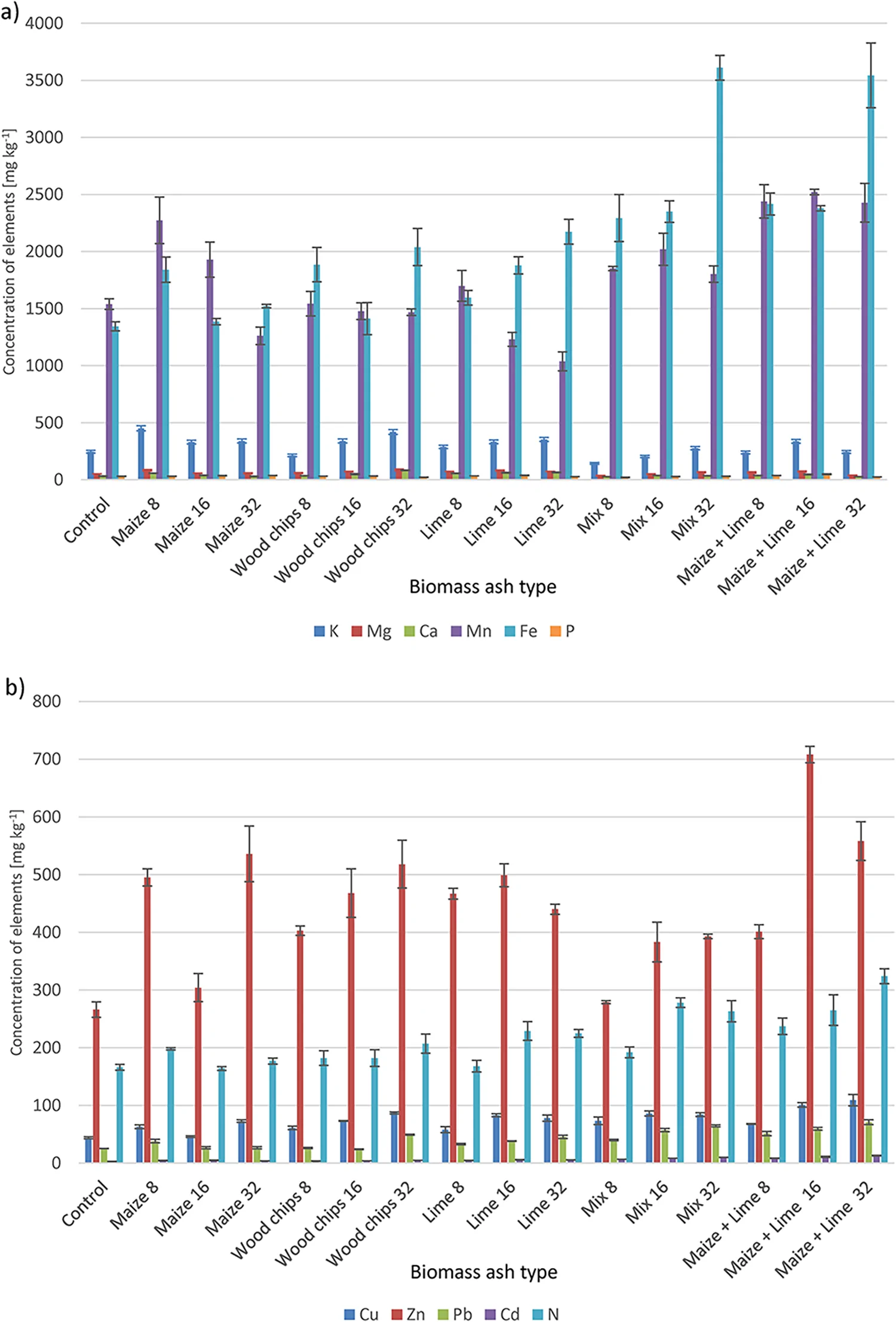
The effect of the biomass ash application on the accumulation of selected elements in leaves of red beet (*Beta vulgaris* L.), (**a**) macronutrients, (**b**) trace elements. Error bars represent standard deviation (*n* = 3).

**Fig. 4:**
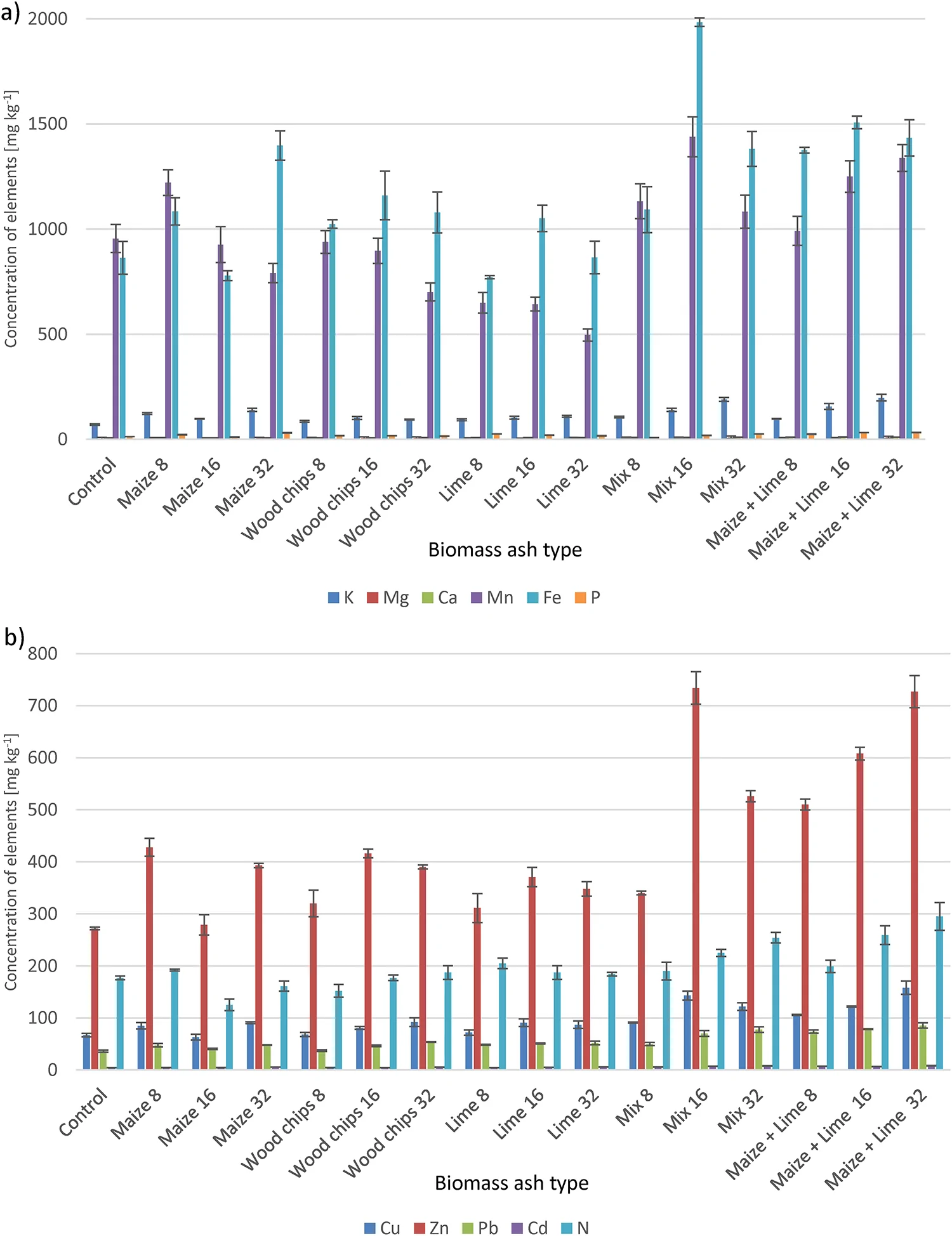
The effect of the biomass ash application on the accumulation of selected elements in roots of red beet (*Beta vulgaris* L.), (**a**) macronutrients, (**b**) trace elements. Error bars represent standard deviation (*n* = 3).

This biomass increase was directly reflected in the mineral composition of beetroot leaves (Fig. 3a, b) and storage roots (Fig. 4a, b). The highest nutrient increases were observed for maize cob ash combined with defecation lime at 8 Mg ha⁻¹ (+ 91%) and 4 Mg ha⁻¹ (+ 71%), whereas the smallest changes were recorded for pure maize cob ash and lime-based ash (+ 7–13%). Mechanistically, this response is consistent with improved soil chemical conditions following liming, where the dissolution of CaCO₃ consumes protons (CaCO₃ + 2 H⁺ → Ca²⁺ + H₂O + CO₂), leading to increased pH and base saturation, and potentially reduced availability of Al³⁺ and Mn²⁺ under acidic conditions, thereby improving root functioning. In addition, co-applied materials may enhance nutrient availability through mineralisation and chelation processes within a more favourable pH range^37^.

Macronutrient distribution showed a clear organ-specific pattern. Leaves (Fig. 3a) consistently accumulated higher concentrations of K, Mg, and Ca than storage roots (Fig. 4a), with leaf-to-root ratios of 3.5 for K, 3.0 for Mg, and 4.9 for Ca in the control, which is typical for *Beta vulgaris*^40^. Across treatments, the K: Mg ratio in leaves increased to ~ 7.5, exceeding the optimal physiological range (3:1–5:1) and indicating K–Mg antagonism. This imbalance suggests competition between cations for uptake sites, as excessive K supply may limit Mg acquisition and affect overall nutrient balance. Similar effects have been reported in plant nutrition studies, where excessive potassium availability can suppress Mg uptake and affect plant physiological functions. On a cellular level, this antagonism occurs because high concentrations of K+ ions in the soil solution competitively inhibit the binding of Mg^2^^+^ to specific transport proteins in the root plasma membrane, effectively suppressing secondary magnesium uptake pathways and triggering downstream disruptions in chloroplast ultrastructure^39,41^.

Root micronutrient enrichment (Zn, Fe, Mn, Cu) was most pronounced after application of blended ashes at both 4 and 8 Mg ha⁻¹ (Fig. 4a, b). Although increased pH may enhance Zn availability, excessive K supply may simultaneously limit micronutrient uptake through competitive interactions^36^. This bioaccumulation pattern is consistent with the leaching behaviour of the applied materials (Table 3), indicating that elements with higher mobility in soil solution can be more readily taken up by plants.

From a food-safety perspective, trace metal concentrations in the edible root are critical (Fig. 4b). The lowest Pb levels were observed for maize cob ash at 4 Mg ha⁻¹ (− 23%), whereas the lowest Cd levels occurred under wood-chip ash (− 2%). In contrast, blended treatments increased Pb and Cd uptake relative to the control. For example, the mixture of maize cob and wood-chip ash at 8 Mg ha⁻¹ increased Pb by 115% and Cd by 82%, although the resulting concentrations remained low.

This pattern is consistent with the potential influence of dissolved organic carbon (DOC), which may increase in blended ash treatments containing higher proportions of agro-industrial residues and promote the formation of soluble metal–organic complexes^13^. While liming typically reduces Cd and Pb availability through pH-driven immobilisation, the addition of more labile organic fractions may enhance DOC and increase metal mobility. Therefore, the elevated Pb and Cd concentrations observed in these blended treatments are likely associated with DOC-mediated transport, which may not be fully offset by alkalisation. This highlights the importance of considering both mineral and organic fractions when assessing ash safety and supports the recommendation to stabilise organic inputs prior to application^42^.

From a regulatory perspective, the EU framework sets a maximum Pb level of 0.10 mg kg⁻¹ fresh weight (FW) for root vegetables, along with strict Cd limits^43,44^. However, these regulatory thresholds are typically based on total metal concentrations and do not account for element mobility and bioavailability. The present results indicate that even when total concentrations remain within permissible limits, increased leaching and DOC-mediated complexation may enhance metal transfer to plants, potentially affecting food safety. This highlights a key limitation of current regulatory frameworks, as they may underestimate environmental risk when assessments do not include mobility-based parameters or plant uptake data. In particular, agro-industrial residues and high-organic blends may pose a greater challenge for compliance due to increased variability in both total content and leaching behaviour. Therefore, the findings of this study support the need for a more comprehensive assessment approach, integrating chemical composition, leaching characteristics and biological response, to better reflect real field conditions and ensure safe agricultural use of biomass ashes. Because the current dataset is expressed primarily on a dry-matter (DM) basis (Figs. 3a and b and 4a and b), interpretation in terms of regulatory thresholds based on fresh weight requires appropriate conversion. However, such assessment remains indicative, as variability in moisture content between plant organs may influence the final value.

Based on the integrated assessment of yield, nutrient uptake, and trace metal accumulation, the individual application of maize ash or wood-chip ash at a rate of 16 g pot⁻¹ (equivalent to 4 Mg ha⁻¹) is recommended as the most sustainable strategy, providing a favourable balance between improved plant growth, nutrient supply, and low trace metal accumulation.

## Conclusions

Among the six biomass ashes evaluated, ashes from distillery decoction and sugar beet pulp exceeded the EU 2019/1009 arsenic limit by up to 5.5-fold and are unsuitable for agricultural use without upstream process modification (e.g., transition to indirect steam drying). These feedstocks should be excluded from soil application until arsenic contamination is eliminated at source.

Maize cob ash and wood-chip ash applied at ~ 4 Mg ha⁻¹ represent the most agronomically safe strategy: they raised soil pH toward the physiological optimum for *Beta vulgaris* L. (pH 6.0–6.8), increased total fresh biomass by up to 59%, and reduced Pb and Cd uptake compared to high-dose blended treatments. Higher doses and organic-rich blends should be avoided, as DOC-mediated complexation at 8 Mg ha⁻¹ increased root Pb by 115% and Cd by 82%, demonstrating that alkalisation alone does not offset DOC-driven metal mobilisation.

Regulatory compliance based on total metal concentrations alone is insufficient. Leaching tests revealed that even ashes within EU permissible limits can release significant proportions of As, Cr, and Zn (up to 85% in the case of Cr from lime-modified wood-chip ash), and DOC-driven metal transport can elevate plant uptake independently of soil pH. Safe agricultural valorisation of biomass ashes therefore requires integrated assessment combining chemical characterisation, standardised leaching tests, and crop bioassays prior to field application.

## Data Availability

All data generated or analysed during this study are included in this published article.
